# Adaptive Matching of High-Frequency Infrared Sea Surface Images Using a Phase-Consistency Model

**DOI:** 10.3390/s25051607

**Published:** 2025-03-06

**Authors:** Xiangyu Li, Jie Chen, Jianwei Li, Zhentao Yu, Yaxun Zhang

**Affiliations:** 1 Qingdao Innovation and Development Center, Harbin Engineering University, Qingdao 266500, China; li_xiangyu@hrbeu.edu.cn (X.L.); zhangyaxun@hrbeu.edu.cn (Y.Z.); 2 Institute of Remote Sensing, Naval Submarine Academy, Qingdao 266000, China; lgm_jw@163.com (J.L.); qyyzt@163.com (Z.Y.)

**Keywords:** sea surface remote sensing image, phase-consistency feature description, adaptive matching algorithm

## Abstract

The sea surface displays dynamic characteristics, such as waves and various formations. As a result, images of the sea surface usually have few stable feature points, with a background that is often complex and variable. Moreover, the sea surface undergoes significant changes due to variations in wind speed, lighting conditions, weather, and other environmental factors, resulting in considerable discrepancies between images. These variations present challenges for identification using traditional methods. This paper introduces an algorithm based on the phase-consistency model. We utilize image data collected from a specific maritime area with a high-frame-rate surface array infrared camera. By accurately detecting images with identical names, we focus on the subtle texture information of the sea surface and its rotational invariance, enhancing the accuracy and robustness of the matching algorithm. We begin by constructing a nonlinear scale space using a nonlinear diffusion method. Maximum and minimum moments are generated using an odd symmetric Log–Gabor filter within the two-dimensional phase-consistency model. Next, we identify extremum points in the anisotropic weighted moment space. We use the phase-consistency feature values as image gradient features and develop feature descriptors based on the Log–Gabor filter that are insensitive to scale and rotation. Finally, we employ Euclidean distance as the similarity measure for initial matching, align the feature descriptors, and remove false matches using the fast sample consensus (FSC) algorithm. Our findings indicate that the proposed algorithm significantly improves upon traditional feature-matching methods in overall efficacy. Specifically, the average number of matching points for long-wave infrared images is 1147, while for mid-wave infrared images, it increases to 8241. Additionally, the root mean square error (RMSE) fluctuations for both image types remain stable, averaging 1.5. The proposed algorithm also enhances the rotation invariance of image matching, achieving satisfactory results even at significant rotation angles.

## 1. Introduction

Sea surface image matching is an important area of research in computer vision and remote sensing. This technique is used to identify, track, and analyze various objects and phenomena in the marine environment, such as vessels, buoys, marine organisms, pollutants, and weather conditions. Advances in satellite and unmanned aerial vehicle (UAV) technologies have significantly improved the ability to capture sea surface images, thus enhancing the importance of image matching in applications like marine monitoring, safety, and environmental protection [1,2,3]. At the same time, due to the susceptibility of the sea surface to weather conditions, lighting, wave activity, and other factors, the use of different methods to capture images also results in variability and complexity in the outcomes, which presents challenges for the matching task.

The results of image matching directly influence subsequent image fusion and stitching processes. The more accurate the matching results, the greater the reduction in misalignment and ghosting issues during subsequent fusion, thereby providing a solid foundation for improving the spatial and radiometric resolution of infrared images. Traditional image-matching techniques often rely on hand-crafted feature extraction algorithms [4,5,6]. Techniques like SIFT (Scale-Invariant Feature Transform) [7] and SURF (Speeded Up Robust Features) [8] effectively extract key points and generate feature descriptors for matching. The Histogram of Oriented Gradients (HOG) method computes the gradient direction histogram to extract shape features, making it particularly effective for object detection and recognition. Color histograms can also analyze the color distribution of an image for matching. However, this method is sensitive to changes in illumination. Various measurement techniques, such as Euclidean distance and Hamming distance, can be used for feature matching. Additionally, RANSAC (Random Sample Consensus) can be integrated to remove false matches and improve matching accuracy [9]. To tackle challenges arising from differences in illumination, contrast, and nonlinear radiation distortion in heterogeneous remote sensing images, a heterosource image-matching method has been proposed. This method incorporates anisotropic weighted moments and absolute phase-consistency direction histograms, proving effective [10]. In deep learning, convolutional neural networks (CNNs) are widely used for image-matching tasks, significantly improving accuracy by autonomously learning feature representations. Integrating depth feature fusion with various depth extraction techniques and applying the Attention Mechanism allows for focused attention on critical image regions, enhancing matching performance. Furthermore, Generative Adversarial Networks (GANs) generate samples through adversarial training, improving the model’s matching capabilities across various sea surface conditions [11,12,13].

Despite advancements in traditional algorithms and deep learning techniques, matching sea surface images remains challenging. Most existing algorithms are designed for matching terrestrial targets, which are typically static and thus easier to identify feature points. Under this premise, we employ a high-frame-rate infrared array camera to image the sea surface. This camera primarily detects in the long-wave infrared band, with the mid-wave infrared band serving as an auxiliary detection band. The dual-band arrangement is positioned in a front-to-back configuration, enabling forward scanning and gaze imaging to capture multi-dimensional information of the target. It offers high-sensitivity imaging in both the long-wave and mid-wave infrared spectral ranges under airborne environmental conditions and is capable of supporting airborne testing and subsequent image-processing tasks. Although the infrared images of the sea surface are captured using a high-frame-rate infrared camera, which largely mitigates the issue of rapid changes on the sea surface, the inherent complexity of the marine environment introduces various factors, such as weather conditions, tidal movements, and wave activity, which continuously alter the roughness of the sea surface. This dynamic nature results in fluctuations in image quality, complicating the identification of feature points on the sea surface. Therefore, developing effective methods for matching, fusing, and stitching feature points across extensive sequences of sea surface images remains an area needing further research.

In a paper published in 1981, Oppenheim et al. pointed out that in the Fourier representation of signals, the spectral amplitude and phase often play different roles. In some cases, significant characteristics of the signal can be retained by preserving only the phase information [14]. Therefore, this paper proposes an algorithm based on the phase-consistency model, which not only focuses on the features of weak texture information on the sea surface, effectively addressing the challenges posed by complex and dynamic sea surface conditions, but also enhances the accuracy and robustness of the overall matching results. Moreover, this algorithm overcomes the limitations of previously proposed algorithms, significantly reducing the matching time and demonstrating adaptability to multimodal matching scenarios, effectively handling different types of images.

(1) We propose an adaptive weighting function that uses local contrast and edge information to dynamically adjust the weight distribution of maximum and minimum moments in the anisotropic weighted moment diagram. This approach tailors the matching strategy to varying image features, improving performance across different structures in the image.

(2) To achieve rotation invariance, we extend the directional characteristics of the derived phase feature consistency to create an omnidirectional phase-consistency directional gradient.

(3) We develop a 328-dimensional log-polar coordinate descriptor using the gradient position and direction histogram algorithm.

## 2. Related Work

Image registration is a method used to align and overlay multiple images taken under different conditions, with various detectors or at different times [15]. Recent technological advancements have continuously improved research methods for matching sea surface images. Today’s sea surface image-matching techniques include both traditional feature extraction methods and advanced deep learning approaches. These methods have significantly improved the accuracy and reliability of image matching [16].

The pixel-based method uses gradient, color, or geometric information from the image to estimate a transformation matrix. This helps to align the transformed image with the original image by reducing discrepancies [17]. Qi Zhi et al. tackled the problem of parallax from camera movement using depth cues and smooth transition criteria, although this method is quite complex [18]. S. Peleg used an optical flow method to create multiple aligned strips, allowing for the development of adaptive manifolds. This method effectively addresses the limitations of other projection techniques that depend on fixed manifolds, helping to prevent cumulative errors. However, the accuracy of the stitching produced remains suboptimal [19]. The previously mentioned methods achieve reasonable registration accuracy when the gray values of the two images are similar and only involve translational transformations. However, when the images have high spatial resolution, the efficiency of this method decreases, limiting its application.

Feature-based matching technology has been widely studied in the literature [20]. Key feature-based methods include the Scale-Invariant Feature Transform (SIFT), Accelerated Robust Features (SURF), Oriented FAST and Rotated BRIEF (ORB), along with various techniques for enhanced feature matching and key point assignments [21,22,23]. These methods are effective for handling images with linear distortion variations and can address image registration challenges from various angles, such as optimizing binary descriptions and ensuring robustness to scale and rotation [20]. Nevertheless, the performance of these algorithms declines in situations with substantial temporal and geometric changes. Additionally, Ye et al. proposed a new image-matching technique that measures similarity based on shape attributes. This method constructs self-similar shape descriptors for effective image matching, but its use is limited due to its dependence on the image’s contour or shape [24]. Recently, a histogram-oriented phase-consistency algorithm was introduced to improve matching in Magnetic Resonance Spectroscopic Imaging (MRSI). Although better results can be obtained, the effectiveness of this method is significantly compromised when the geographic information is inaccurate. [25]. Zhu et al. suggested a multimodal matching method that integrates repeatable detectors and rotation-invariant descriptors [26]. Furthermore, Ye et al. created a new Scale-Invariant Feature Descriptor (SFOC) using first- and second-order Gaussian steerable filters, although this descriptor is sensitive to global geometric distortions between images [27].

In recent years, advancements in computing power and data availability have accelerated the development and widespread application of deep learning technology [28]. Deep learning methods now replace several steps in traditional feature-based approaches, including feature point extraction, descriptor calculation, similarity measurement, correspondence matching, and outlier removal [29]. Ji et al. introduced a registration method leveraging deep convolutional neural network feature fusion [30]. SIFNet is an algorithm that employs self-focused interactive fusion networks [31]. Zhang et al. developed a self-supervised training network designed to detect optimal key points [32]. Li et al. introduced the Cross-Mode Matching Network (CM-Net) [33]. Li et al. also proposed an end-to-end framework featuring self-focused and double-supervised losses [34]. Xu et al. presented the local descriptor SODescNet [35]. Ji et al. assessed both traditional and deep learning-based methods, noting that deep learning approaches still have room for improvement [30]. These methods apply deep learning to parts of multi-modal image matching, progressively achieving end-to-end unsupervised matching and enhancing matching techniques over time.

In summary, although researchers have made significant strides in image matching, current methods still face notable limitations. Pixel-based methods utilize detailed image information for registration but are computationally intensive, making it challenging to meet real-time processing demands. While feature-based matching has yielded some success, it faces several issues including high computational time, lack of support for geometric transformations, limited image structure information, insufficient key point extraction, and high computational cost. Deep learning-based image-matching methods have improved matching accuracy but continue to struggle with the challenge of acquiring large-scale annotated data sets. Furthermore, the difficulty in obtaining sufficient training samples means that these methods require further study to address computational complexity and generalization issues. To address these limitations, this paper proposes a robust image-matching algorithm based on the phase-consistency model, which effectively overcomes the shortcomings of previous methods: (1) It significantly reduces matching time, meeting real-time processing requirements; (2) Testing shows that the algorithm identifies a higher number of corresponding points than other methods, improving image-matching accuracy; (3) It adapts to multi-modal matching scenarios and handles various image types effectively; (4) Its strong rotational invariance enables it to achieve effective matching even when the target image has a significant rotation.

## 3. Method

**Figure 1:**
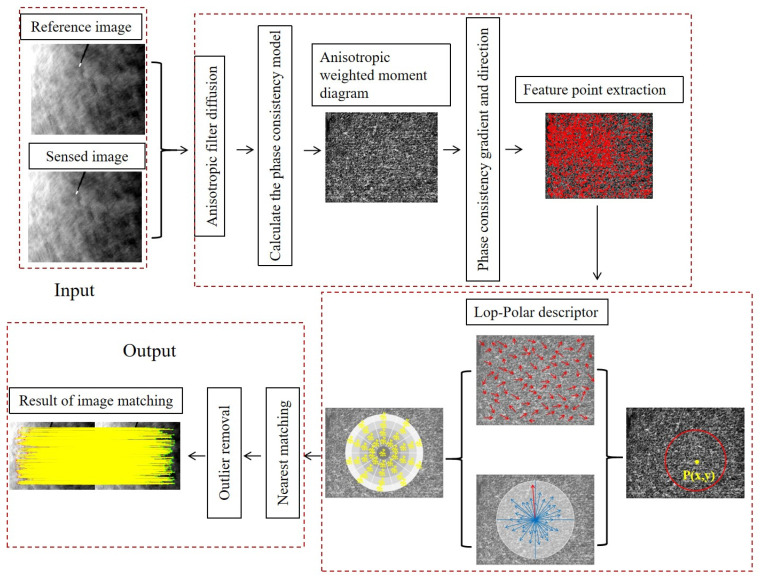
Workflow of matching algorithm in this paper.

In this study, we apply the phase-consistency model for matching sea surface images in the frequency domain. The process of the matching algorithm is illustrated in Figure 1: (1) First, we construct a nonlinear scale space using the nonlinear diffusion method. The Log–Gabor odd-symmetric filter in the two-dimensional phase-consistency model produces the maximum and minimum moments. We then construct the anisotropic weighted moment equation and compute the corresponding diagram. (2) Next, we identify the extremum points in the anisotropic weighted moment space. (3) Phase-consistency values serve as image gradient features, and Log–Gabor-based feature descriptors, robust to scale and rotation changes, are generated. (4) Using Euclidean distance as a similarity metric for initial matching, the feature descriptors are compared, and mismatches are filtered out using the Fast Sample Consensus (FSC) algorithm.

### 3.1. Construction of Scale Space Using Nonlinear Filtering

In image matching and feature extraction, we begin by constructing a nonlinear scale space using a nonlinear diffusion method, which generates a multi-scale representation through adaptive image smoothing. This process is derived from classical linear scale-space theory but substitutes linear diffusion with nonlinear diffusion to better handle complex image structures. Using an optimized nonlinear diffusion approach, the anisotropic diffusion equation is defined as follows [36]:

(1) $$\frac{\partial L}{\partial t}=div\left[\frac{1}{1+{\left|\nabla L_{\sigma }\right|}^{2}/k^{2}}\cdot \left(x,y,t\right)\nabla L\right]$$

(2) $$L^{n+1}=\frac{L^{n}}{2\left(I-2\tau A_{1}\left(L^{n}\right)\right)}+\frac{L^{n}}{2\left(I-2\tau A_{2}\left(L^{n}\right)\right)}$$

where div represents the divergence operator, t represents the scale value of the time measure, $\frac{1}{1+{\left|\nabla L_{\sigma }\right|}^{2}/k^{2}}$ is the diffusion coefficient, k is the contrast factor, L is the difference image, and I is the current image. $A_{1}\left(L^{n}\right)$ and $A_{2}\left(L^{n}\right)$ represent the row direction and column direction diffusion coefficient matrix of coded image $L^{n}$, respectively. The final result of diffusion is denoted as $L^{n+1}$, and τ indicates the time step of diffusion. During the diffusion calculation, the scale value is converted to time, that is $t_{n}=1/2\sigma_{n}^{2}$, $\sigma_{n}$ represents the scale, the number of layers belongs to $\left[0,1,2,\cdots ,S\right]$, and S represents the maximum number of layers.

This model effectively preserves image structures in high-gradient areas (e.g., edges and details) by adjusting the diffusion coefficient, while achieving smoother results in low-gradient regions (e.g., flat areas). As scale space is constructed, gradual image smoothing over time creates representations at various scales. The nonlinear scale space is ideal for detecting multi-scale features and enhances feature-matching robustness by providing smooth, structure-preserving representations at all scales, reducing noise interference. Compared to linear diffusion, nonlinear diffusion better preserves structural information, making it more effective for images with complex edges and textures. This improves the accuracy of image matching and increases resistance to interference.

### 3.2. Phase-Consistency Model Calculation

In earlier studies, Kovesi optimized the phase-consistency model by extending it from one-dimensional signals to two-dimensional images, proposing its application for edge detection [37]. The model uses a multi-scale, multi-direction two-dimensional Log–Gabor filter to convolve the image. The Log–Gabor function has a Gaussian frequency response on the logarithmic frequency axis, allowing for flexible bandwidth filter construction while preserving zero DC components in even symmetric filters. This filter is then convolved with the signal to compute phase consistency. By calculating the local energy of the image, a two-dimensional phase-consistency model is generated. This approach is resistant to changes in local illumination and effectively extracts stable edge information in complex backgrounds.

The Log–Gabor function is expressed as follows [38]:

(3) $$L\left(\omega ,\theta \right)=\exp\left\{-\frac{{\left[In\left(\omega /\omega_{0}\right)\right]}^{2}}{2{\left[In\left(\sigma /\omega_{0}\right)\right]}^{2}}\right\}\exp\left[\frac{-{\left(\theta -\theta_{0}\right)}^{2}}{2\sigma_{\theta }^{2}}\right]$$

where $\omega_{0}$ is the center frequency of the filter, σ is the bandwidth coefficient, $\sigma /\omega_{0}$ is a constant, $\theta_{0}$ is the filter direction, and $\sigma_{\theta }$ is the standard deviation of the Gaussian function.

The Log–Gabor filter is constructed in the frequency domain, and its spatial domain counterpart is obtained using the inverse Fourier transform:

(4) $$L\left(x,y,s,o\right)=L^{even}\left(x,y,s,o\right)+iL^{odd}\left(x,y,s,o\right)$$

where the real part $L^{even}\left(x,y,s,o\right)$ represents an even symmetric Log–Gabor wavelet, and the imaginary part $L^{odd}\left(x,y,s,o\right)$ represents an odd symmetric Log–Gabor wavelet, s is the scale, and o is the direction.

The two-dimensional image $I\left(x,y\right)$ is convolved separately with the even-symmetric and odd-symmetric Log–Gabor filters. The response values $e_{s,o}\left(x,y\right)$ and $o_{s,o}\left(x,y\right)$ of the image under two filters are obtained.

(5) $$\left[e_{s,o}\left(x,y\right),o_{s,o}\left(x,y\right)\right]=\left[I\left(x,y\right)*L^{even}\left(x,y\right),I\left(x,y\right)*L^{odd}\left(x,y\right)\right]$$

The amplitude $A_{s,o}\left(x,y\right)$ and phase $\phi_{s,o}\left(x,y\right)$ at scale s and direction o are then calculated:

(6) $$A_{s,o}\left(x,y\right)=\sqrt{e_{s,o}^{2}\left(x,y\right)+o_{s,o}^{2}\left(x,y\right)}$$

(7) $$\phi_{s,o}\left(x,y\right)=\arctan\left[\frac{o_{s,o}\left(x,y\right)}{e_{s,o}\left(x,y\right)}\right]$$

The two-dimensional phase-consistency model is computed as follows:

(8) $$PC\left(x,y\right)=\frac{\sum_{s}\sum_{o}\omega_{0}\left(x,y\right)\left\lfloor A_{s,o}\left(x,y\right)\Delta \Phi_{s,o}\left(x,y\right)-T\right\rfloor }{\sum_{s}\sum_{o}A_{s,o}(x,y)+\xi }$$

where $\omega_{0}\left(x,y\right)$ is the weight factor for frequency expansion, $\left\lfloor \cdot \right\rfloor$ prevents a negative value, T is the noise threshold, ξ is the minimum value that prevents the denominator from being zero, and $\Delta \Phi_{s,o}\left(x,y\right)$ is the two-dimensional phase-deviation function [39].

The phase-consistency model derived from the above formula is commonly used for feature extraction in image processing. This model preserves the structural integrity of images in the frequency domain, ensuring consistent feature representation. The weighted moment map incorporates frequency domain features to compute weighted moments in image processing. This approach effectively captures the geometric features of images, enhancing the detection and analysis of crucial structural elements.

### 3.3. Construction of Anisotropic Weighted Moment Diagram

In image processing, “moment” refers to the description of an image’s geometric features, such as shape distribution, position, and orientation. By calculating these moments, geometric properties like center position, rotation angle, and shape complexity can be determined. Weighted moment maps add weights to these calculations, emphasizing key features within the image. The weighted moment diagram is calculated by incorporating phase consistency in multiple directions and edge information, ensuring that directional variations are effectively captured. Based on the moment analysis algorithm, the primary axis corresponds to the direction with the minimum moment, while the perpendicular axis aligns with the maximum moment, indicating key structural features. The magnitude of these moments reflects the distinctiveness of a feature point. A high minimum moment indicates a corner feature, which is derived using the following formula to determine the maximum and minimum moments of phase consistency:

(9) $$A={\sum_{o}\left(P\left(\theta_{o}\right)\cos\theta_{o}\right)}^{2}$$

(10) $$B=2\sum_{o}\left(P\left(\theta_{o}\right)\cos\theta_{o}\right)\times \left(P\left(\theta_{o}\right)\sin\theta_{o}\right)$$

(11) $$C={\sum_{o}\left(P\left(\theta_{o}\right)\sin\theta_{o}\right)}^{2}$$

(12) $$M=\frac{1}{2}\left(C+A+\sqrt{B^{2}+{\left(A-C\right)}^{2}}\right)$$

(13) $$m=\frac{1}{2}\left(C+A-\sqrt{B^{2}+{\left(A-C\right)}^{2}}\right)$$

(14) $$\Phi =\frac{1}{2}\arctan\left(\frac{B}{A-C}\right)$$

Here, *M* represents the maximum moment of phase consistency, *m* is the minimum moment, and Φ is the spindle angle, which defines the directional characteristic of phase consistency. *A*, *B*, and Care intermediary values in the calculation of the moments.

In this study, an adaptive weighting function is employed to create an anisotropic weighted moment diagram. The weights are dynamically adjusted based on the local differences between the maximum and minimum phase-consistency moments and other image features. When the local difference between the maximum and minimum moments is large, it indicates strong anisotropic characteristics, prompting an increase in weight for that region. Conversely, a small difference indicates a uniform region, where the anisotropic weight is reduced. We design an adaptive weighting function to allow weights to be adjusted dynamically based on local characteristics.

(15) $$ct=\frac{M-m}{M+m+\xi }$$

(16) $$W_{a}(ct)=\frac{1}{1+e^{-\alpha (ct-\beta )}}$$

In this function, *M* is the maximum moment and m is the minimum moment. The constant ξ ensures numerical stability by preventing a zero denominator. The parameter *ct* represents the contrast, while α controls the slope of the function—the larger the α, the sharper the transition. β defines the central value, with the most significant weight changes occurring where the contrast is near β.

An edge-detection algorithm is also applied to capture edge information, serving as an additional constraint on the weighted factors. This enhances contrast response in edge regions, ensuring that key features are well-defined while maintaining smoothness in non-edge areas.

(17) $$W_{e}\left(ct\right)=W_{a}\left(ct\right)+\gamma \cdot E$$

Here, E represents edge information, and γ denotes the weight factor, expressed as:

(18) $$M_{weight\_\mathrm{new}}=W_{e}(ct)\cdot M+\left(1-W_{e}(ct)\right)\cdot m$$

In this manner, the weight adapts to local contrast variations, enhancing regions with strong anisotropy and applying smoother adjustments to uniform regions. When contrast is high (indicating a large difference between maximum and minimum moments), the weighting function emphasizes the maximum moment to highlight dominant structures. When contrast is low, the moment diagram aligns more closely with the average of the maximum and minimum moments, indicating uniformity.

**Figure 2:**
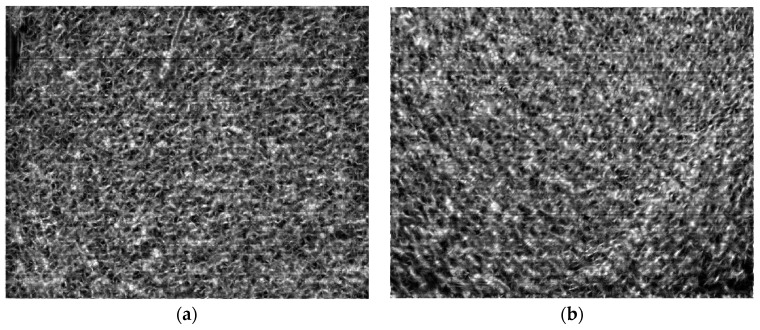
The anisotropic weighted moment map: (**a**) Long-wave infrared image; (**b**) Medium-wave infrared image.

In the process of image matching, we conducted matching tests for both infrared long-wave and mid-wave images. Figure 2 presents the anisotropic weighted moment map of a reference image, with the left image representing the infrared long-wave image and the right image representing the infrared mid-wave image. From the images, it is evident that the texture features and edge details of the sea surface are more effectively highlighted.

### 3.4. Detection of Feature Points

**Figure 3:**
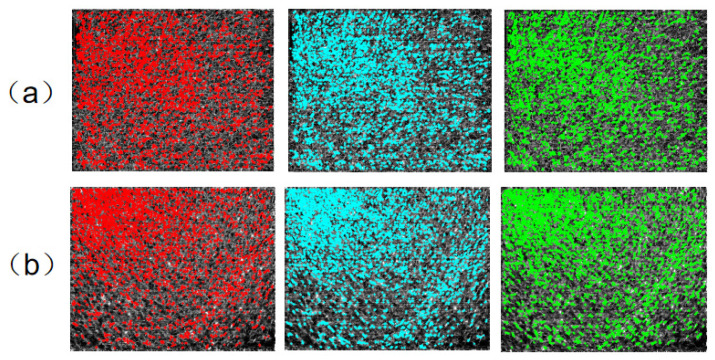
Feature point detection results of anisotropic weighted moment diagram. (**a**) long-wave infrared image (**b**) medium-wave infrared image.

**Figure 4:**
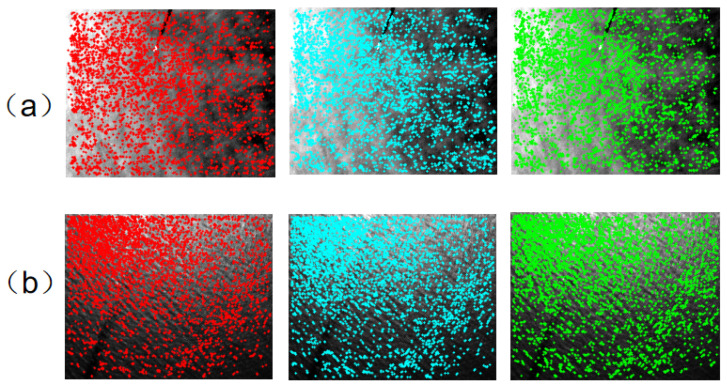
Feature point detection results of the original image. (**a**) long-wave infrared image (**b**) medium-wave infrared image.

Phase consistency is a frequency-domain-based feature detection method. The minimum moment m corresponds to corner detection, while the maximum moment M outlines the edges in the image. Edge structures are highly resistant to radiometric distortions, improving the accuracy of corner and edge feature detection. We employ the Harris algorithm for corner detection and the Features from Accelerated Segment Test (FAST) operator for edge feature detection. After iterative detection, filtered results are retained as feature points. Figure 3 displays the feature point detection results for both long-wave and medium-wave infrared images, based on the anisotropic weighted moment diagram, and Figure 4 shows the corresponding original image detection outcomes. The three different colors indicate different points in time when the image was acquired.

### 3.5. Feature Description and Matching

Creating feature descriptors using the Log–Gabor filter is a crucial step in image processing. This method produces descriptors that are robust to changes in scale and rotation by leveraging the filter’s frequency-domain properties. Using the two-dimensional phase-consistency model, we transform the phase-consistency features into gradient features to extract key image information. The phase-consistency directional features are constructed using the odd-symmetric filter of the Log–Gabor function, as shown in Equation (14) above. The results of the odd-symmetric filter are projected in multiple directions onto horizontal and vertical components, yielding horizontal energy *A* and vertical energy *C*. To ensure rotation invariance, we extend the directional phase-consistency features to create an omnidirectional phase-consistency gradient, calculated as follows:

(19) $$A=\left\{\begin{array}{l} \frac{\Phi *180}{\pi }\;,\;\Phi \left[i\right]\left[j\right]>0\\ \Phi +2\pi \;,\;\Phi \left[i\right]\left[j\right]<0 \end{array}\right.$$

For each feature point, a circular neighborhood is selected, centered on the feature point. We extract the phase amplitude and directional consistency for the pixels in the neighborhood, build a feature vector, and generate a gradient histogram of the phase-consistency direction based on these statistics. The gradient histogram spans [0, 360] degrees, divided into 36 bins, each covering 10°. The gradient and direction characteristics of each bin are then computed. Each neighborhood is weighted based on distance from the center point, with farther neighborhoods contributing less to the histogram. The peak of the histogram defines the principal direction of the feature points.

**Figure 5:**
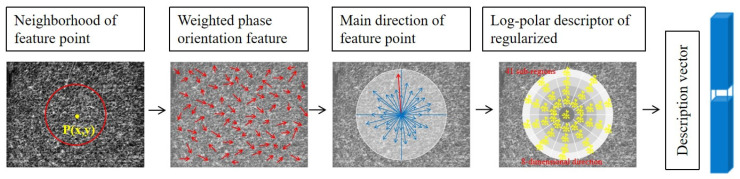
Descriptor generation flowchart.

After identifying the principal direction, we use a log-polar frame to build descriptors [40]. The circular neighborhood is divided into 41 equal-area subregions, forming an antipolar coordinate grid. For each subregion, eight directional gradients are computed. A 328-dimensional feature vector is generated, effectively capturing the image’s features and enhancing the accuracy of image matching. Figure 5 illustrates the entire process of descriptor generation.

We use Euclidean distance as the similarity metric for initial matching, identifying a preliminary set of matching points. As this set may contain mismatches, we apply the FSC algorithm to refine the matches and remove incorrect points. Thus, even in the presence of noise and variations in image conditions, we are able to identify a set of accurate and reliable correspondences.

## 4. Experimental Results

To evaluate the effectiveness of the proposed algorithm, we compared it against traditional methods such as SIFT, SURF, ORB, and HAPCG. Based on prior research and testing, we set the feature-extraction threshold to 0.5, the image scale difference to 1.6, and the neighborhood window size to 42 pixels. Parameters for other algorithms were optimized accordingly. Affine transformation was applied as a constraint, with matches considered correct if the error was within three pixels. At least four corresponding points were required for a valid match. We evaluated the algorithm by comparing the number of correct matches (NCM) and root mean square error (RMSE) to assess its strengths and limitations.

(20) $$RMSE=\sqrt{\frac{\sum_{i=1}^{N}{\left(x_{i}-{x_{i}}^{\prime \prime }\right)}^{2}+{\left(y_{i}-{y_{i}}^{\prime \prime }\right)}^{2}}{N}}$$

Here, *N* denotes the number of matching points, $\left(x_{i},y_{i}\right)$ are the coordinates of these points in the reference image and target image, while $\left({x_{i}}^{\prime \prime },{y_{i}}^{\prime \prime }\right)$ represents the transformed coordinates of the same points in the first matched image.

### 4.1. Image Data Sets

The image data for this study were collected from a specific sea area using high-resolution, dual-band infrared cameras mounted on an aerial platform. The cameras captured images in the medium-wave and long-wave infrared ranges, with a frame rate of 50 frames per second and short integration times. The long-wave band (7.7–10.5 μm) served as the primary detection channel, while the medium-wave band (3.3–4.3 μm) acted as the auxiliary channel. The respective array sizes were 640 × 512 for the long-wave and 1280 × 1024 for the medium-wave. Over 1 min, we captured 2372 long-wave and 1187 medium-wave images, which were then used to validate the algorithm developed in this study.

**Figure 6:**
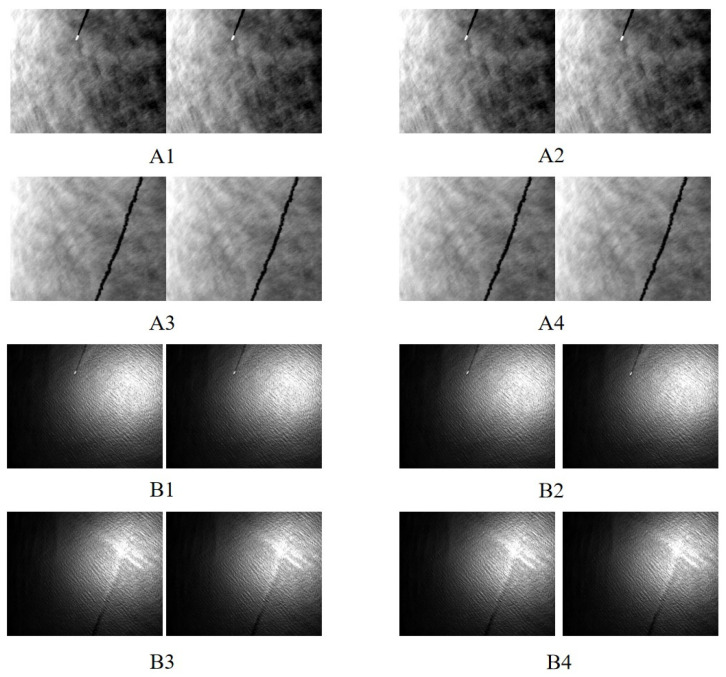
Part of remote sensing images.

Due to significant differences in pixel resolution, both long-wave and medium-wave images were analyzed. The eight-class data image set used in this study contains displacement differences, band differences, etc. Figure 6 presents a subset of remote sensing images. Specifically, Group A consists of long-wave infrared images, while Group B consists of mid-wave infrared images. For the odd-numbered groups, the two images to be matched are adjacent frames, while for the even-numbered groups, the two images are separated by a 10-frame difference. Additionally, the first two image pairs in each group contain sea surface targets, while the last two do not. In this way, a control group setup is established. The displacement between adjacent frames was calculated to be 4.5 m, based on the drone’s speed and altitude.

### 4.2. Qualitative Evaluation

**Figure 7:**
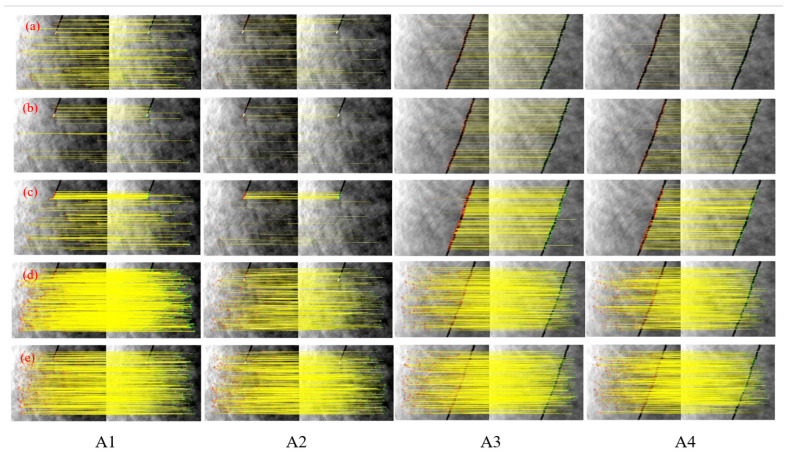
Matching results of long-wave infrared images based on five methods. (**a**) SIFT; (**b**) SURF; (**c**) ORB; (**d**) HAPCG; (**e**) Textual algorithm.

**Figure 8:**
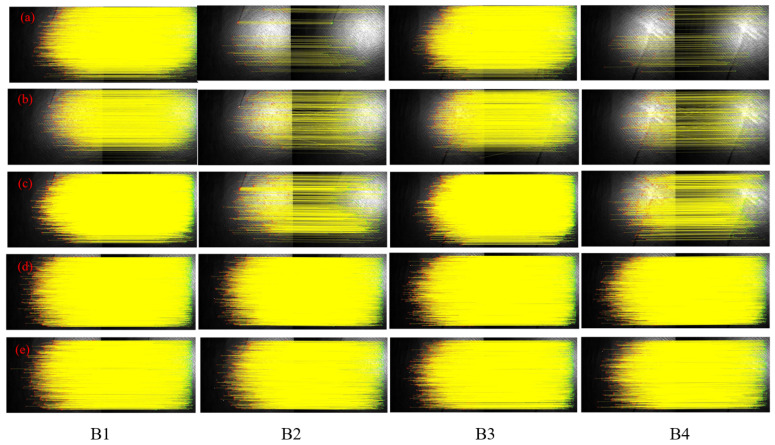
Matching results of medium-wave infrared images based on five methods. (**a**) SIFT; (**b**) SURF; (**c**) ORB; (**d**) HAPCG; (**e**) Textual algorithm.

To assess the algorithm’s effectiveness and robustness, we compared it with established methods such as SIFT, SURF, ORB, and HAPCG. Tests were conducted using both long-wave and medium-wave infrared images, including those with sea surface targets and those with a pure sea surface background. The results are summarized in Figure 7 and Figure 8, which depict matching outcomes for long-wave (Group A) and medium-wave (Group B) images, respectively. Overall, the proposed method outperforms traditional algorithms in both infrared bands, demonstrating superior matching accuracy.

Figure 7 presents the results for the five methods tested on long-wave images (A1 to A4). Figure 7a–c shows the results of the SIFT, SURF, and ORB methods, respectively. In cases with large displacement differences, fewer corresponding points are detected, with feature points concentrated on targets or wake features while ignoring weak sea-surface textures. The performance of these methods is notably weaker than that of the latter two algorithms. Figure 7d shows that HAPCG handles scale and small rotation differences well but struggles with large rotations. The proposed algorithm, shown in Figure 7e, achieves a high NCM detection rate.

Figure 8 shows the results of the five methods applied to medium-wave images (A1 to A4). Compared to the long-wave band, logarithmic matching shows significant improvement. Figure 8a–c displays the results of the SIFT, SURF, and ORB methods. The number of corresponding points decreases in images with large displacement differences, with SURF showing notable errors under these conditions. Wake features in the medium-wave band are less distinct than in the long-wave band, with matching results focused primarily on the sea surface background. Figure 8d,e illustrates the results of HAPCG and the proposed algorithm. Due to the large image size and high NCM values, visual differences are minimal, necessitating further quantitative analysis.

**Figure 9:**
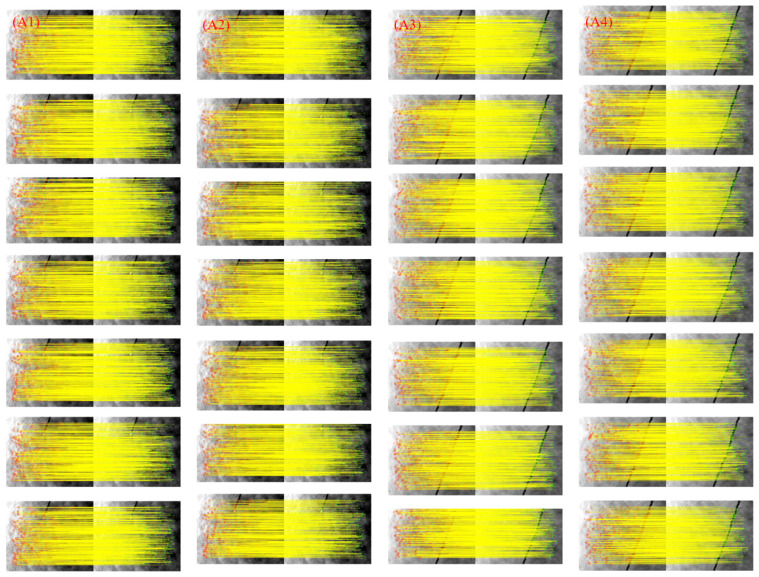
Matching results of long wave based on the textual algorithm.

**Figure 10:**
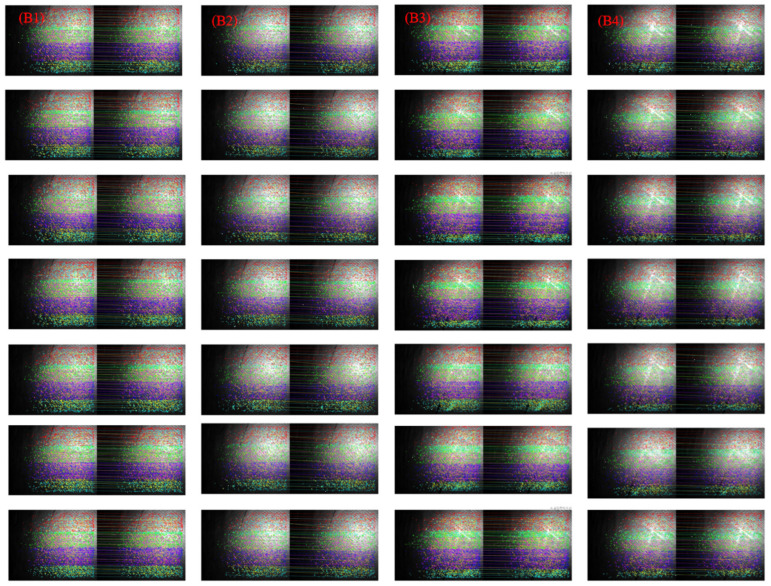
Matching results of medium wave based on the textual algorithm.

Figure 9 and Figure 10 further illustrate the matching results from the data set, showcasing the performance of the proposed method. The proposed algorithm was tested multiple times on the four types of images with distinct differences in Group A and Group B. Figure 9 presents the results for the long-wave image set, while Figure 10 covers the medium-wave set. The figures demonstrate that the proposed method delivers consistent and stable image matching, particularly regarding band and displacement differences. These results suggest that the method can serve as a reliable reference for future image-matching challenges.

As mentioned earlier, due to the larger size of the mid-wave images, when applying the algorithm in this study for image detection, an excessively high number of NCMs are detected, with some even reaching the magnitude of 10,000. When all the matched points and matched lines are displayed on the image, they almost cover 90% of the image, thereby affecting the evaluation results. To resolve this, we implemented a display protocol where all matching points are retained, but matching lines are limited. Images are divided into six color-coded groups, with one matching line shown for every 80 groups, maintaining alignment between line colors and their corresponding points. This method provides a clear visualization of the algorithm’s performance (Figure 10) while preserving critical image features.

### 4.3. Quantitative Evaluation

**Figure 11:**
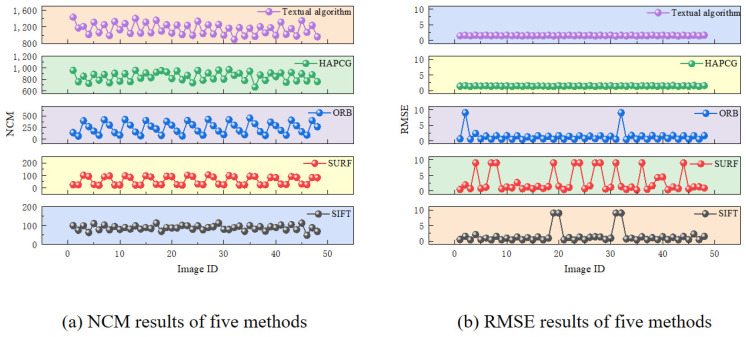
Results of several indicators of long wave.

**Figure 12:**
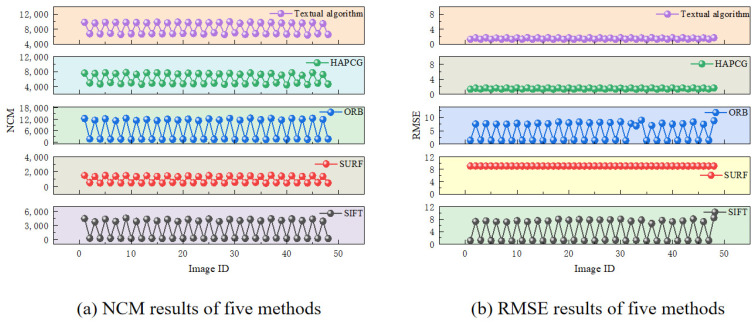
Results of several indicators of medium wave.

The effectiveness of the matching process is measured by the number of matching points, NCM, and Root Mean Square Error (RMSE). Matches with an RMSE exceeding 9 pixels are classified as failed. Figure 11 and Figure 12 show the evaluation results for various performance indicators across different bands. We define the long-wave images A1–A4 and mid-wave images B1–B4 as one set, respectively. Under the same control conditions, 12 test sets are prepared for each group. Therefore, the long-wave and mid-wave images will each display 48 sets of results.

For long-wave images, as shown in Figure 11, the SIFT algorithm achieves a 91.7% matching success rate, with an average RMSE of 0.92. When a sea surface target is present, displacement differences affect performance. The NCM for adjacent frames is 25 higher than for those with a 10-frame difference, and the RMSE is lower. Without a target, the NCM hovers around 90, and some matches fail. When wake features are prominent, SIFT tends to prioritize these features over the sea surface background.

The SURF algorithm underperforms compared to SIFT, with an NCM and RMSE yielding suboptimal results. Its overall success rate is just 75%. When a target is present, SURF detects an NCM of about 25, with better performance for smaller displacement differences. Without a target, the NCM rises to 90, but the success rate drops significantly, and matching failures increase. SURF tends to focus on the target and wake features, regardless of the presence of a target or pronounced wake. If the target’s path covers a large area, the NCM increases, reflecting SURF’s difficulty in accurately matching weak-textured sea surfaces, which affects both accuracy and stability.

The ORB algorithm, leveraging FAST corner detection and BRIEF descriptors, demonstrates high efficiency, with a much faster computation speed than SIFT and SURF. The average NCM for ORB was around 292, and its success rate was 95.8%. The presence of a target greatly impacts detection results. As displacement increases, its effect becomes more noticeable: the NCM for adjacent frames is nearly double that of frames with a 10-frame difference (168 and 405 vs. 81 and 292). The RMSE for adjacent frames is around 0.52 pixels, while for images 10 frames apart, it is 1.6 pixels.

As shown in Figure 11, the NCM values for the three algorithms are relatively low, and the RMSE results fluctuate considerably. This may be due to their limited ability to handle variations in image proportions. The HAPCG algorithm performs well, with an average NCM of 850 and an RMSE of 1.47 pixels. However, HAPCG is effective only when images have small rotational differences, and its performance declines with large rotational discrepancies.

The algorithm proposed in this paper produced the most reliable results, successfully matching all 48 long-wave infrared image pairs. It achieved an average NCM score of 1148 and a 100% success rate. The average RMSE was 1.53 pixels, meeting the target of less than two pixels while significantly improving the NCM over previous methods. Overall, displacement differences significantly affect the algorithm, with an NCM variance of approximately 200. However, the presence of sea surface targets has a minimal impact on the test results, suggesting that the algorithm effectively extracts weak texture features from the sea surface and emphasizes finer detail, thereby enhancing overall accuracy.

Now, we turn to the results for medium-wave images. Figure 12 shows SIFT’s performance, which excels at handling changes in illumination, noise, and affine transformations, while detecting stable feature points. However, displacement differences notably impact SIFT in medium-wave images. For small displacement differences, the mean NCM was 4157 with a RMSE of 1.13. In contrast, for large displacement differences, the mean NCM decreased to 230 with an RMSE of 7.58, indicating that the presence of a target on the sea surface is negligible in this scenario.

In contrast, the SURF algorithm showed minimal sensitivity to displacement differences, as depicted in Figure 12a. The average NCM for adjacent frames was approximately 1400, while frames differing by 10 units had an average NCM of about 512, lower than those obtained by the Scale-Invariant Feature Transform (SIFT). As shown in Figure 12b, testing in the medium-wave band resulted in an RMSE exceeding nine pixels, highlighting significant mismatches within data groups. Therefore, SURF is not an ideal algorithm for medium-wave band detection.

The ORB algorithm was used to detect images in the medium-wave band. Figure 12 illustrates that the presence of a target on the sea surface has a negligible effect on the results. However, displacement difference significantly impacts performance among the five analyzed algorithms. The NCM for adjacent frames is 7.7 times greater than for frames differing by 10. With small displacement differences, the mean NCM is 11,925, with an RMSE of 1.55. In contrast, for large differences, the mean NCM drops to 1548, and the RMSE increases to 7.8, indicating that although few groups fail to match, the RMSE is still notably high.

The HAPCG algorithm shows strong performance, with minimal impact from the presence of a target on the sea surface. The mean NCM for adjacent frames is 4792, with an RMSE of 1.3. For frames differing by 10, the mean NCM rises to 7509, while the RMSE increases to 1.6. The proposed algorithm performs comparably to HAPCG but achieves a higher NCM. Specifically, the average NCM for adjacent frames is 6727, and for frames with a difference of 10, it is 9756.

Table 1 presents the statistical results of the five methods applied to all image data sets, with the values representing the averages of all results. The table includes the statistics for NCM and RMSE, as detailed below:

In summary, the five algorithms differ mainly in performance. For long-wave images, SIFT works the least, and our algorithm works the best. The presence or absence of targets on the sea surface (comparison between A1, A2 and A3, A4) significantly influences the detection results of all five algorithms. When there are no targets on the sea surface, feature points are more easily detected. For mid-wave images, SURF works the least. Displacement differences (comparison between B1 and B2, B3 and B4) have a greater impact on the detection results of the five algorithms. The smaller the displacement difference, the easier it is to detect feature points. However, whether for long-wave or mid-wave images, the algorithm proposed in this paper demonstrates excellent matching performance in terms of detection results. The NCM detection results show a significant advantage, and the RMSE consistently remains below two pixels, indicating excellent robustness and stability, with good performance in areas such as band differences and displacement differences.

Thus, by increasing the number of matching feature points, we have enhanced the matching accuracy. When applying this algorithm to match high-frame-rate infrared sea surface images, we can significantly reduce the matching error rate. This provides a strong foundation for subsequent image fusion and stitching tasks. The fusion of accurately located feature points enhances the radiometric resolution of infrared images. Furthermore, when dealing with large data sets, this approach effectively reduces the sea surface ghosting issue caused by fusion, thereby improving the spatial resolution of the stitched images.

## 5. Discussion and Analysis

### 5.1. Parameter Settings

To assess the robustness of the proposed algorithm, we analyzed its performance with various parameter settings. Key parameters include scale values, the number of scale space layers, feature point extraction thresholds, and neighborhood window sizes. The threshold for feature point extraction differs by image type; for synthetic aperture radar (SAR) images, it is set at 0.3, while for infrared images, it is adjusted to 0.5. After conducting tests and reviewing existing research, we finalized the feature point extraction threshold at 0.5, the scale difference at 1.6, and the neighborhood window size at 42 pixels. In generating the anisotropic weighted moment diagram using the adaptive weighting function, we set the slope parameter to 10.9 and the control center value to 0.5. Test results show that these parameters produce the highest NCM, confirming the robustness of the algorithm. All algorithm tests in this experiment were done using MATLAB R2024a. The experiments were conducted on an 11th Gen Intel^®^ Core™ i5-1135G7 processor at 2.40 GHz, equipped with 16 GB of RAM, and running the Windows 11 x64 operating system.

### 5.2. Rotation Invariance Analysis

**Figure 13:**
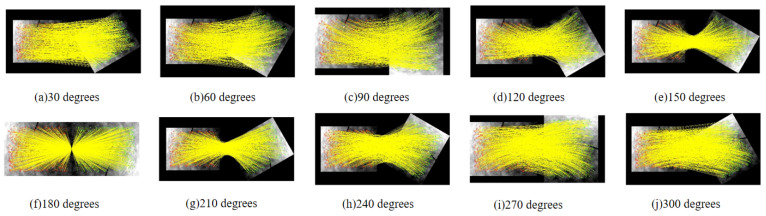
Matching results under different rotation differences of the textual algorithm. (**a**) 30 degrees; (**b**) 60 degrees; (**c**) 90 degrees; (**d**) 120 degrees; (**e**) 150 degrees; (**f**) 180 degrees; (**g**) 210 degrees; (**h**) 240 degrees; (**i**) 270 degrees; (**j**) 300 degrees.

The proposed algorithm not only exhibits enhanced robustness but also achieves high matching accuracy for image groups with considerable rotation differences. We tested it using a set of images with large rotation variations, referencing a long-wave infrared image. This resulted in 11 groups of images with rotation differences from 0° to 360° at 30° intervals. The algorithm was employed to match these images, with results illustrated in Figure 13.

**Figure 14:**
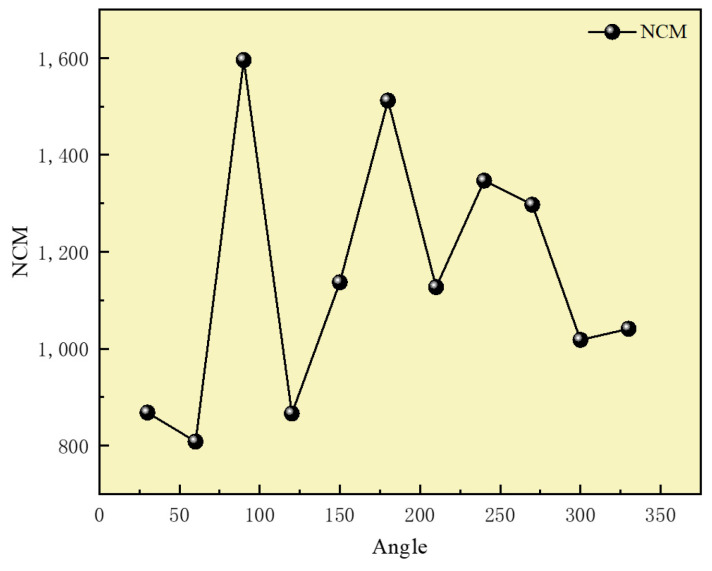
Result of NCM of the rotated image.

**Figure 15:**
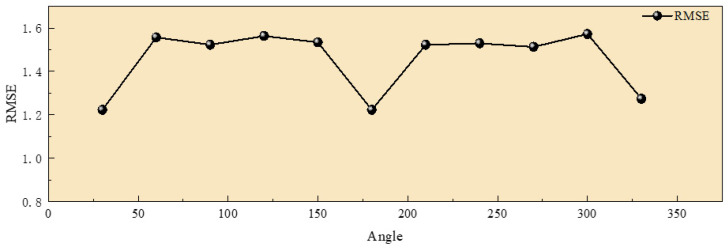
Result of RMSE of the rotated image.

According to the test results, it can be observed that as the rotation angle changes, the algorithm still demonstrates excellent matching performance. The NCM value fluctuates around 1200, and the matching success rate remains high, with the RMSE staying below two pixels, as shown in Figure 14 and Figure 15. Overall, the proposed algorithm significantly enhances rotation invariance in image matching, achieving favorable results even with large rotation angles. In comparison, it outperforms traditional methods in robustness.

## 6. Conclusions

This paper introduces a robust matching method that effectively addresses the challenges of accuracy and rotation invariance in image matching, leading to stable and effective outcomes. We employ a phase-consistency model for matching sea surface images in the frequency domain. An adaptive weighting function, based on local contrast and edge information, dynamically adjusts the matching strategy to accommodate varying image features, thereby enhancing performance across different structures. To ensure rotation invariance, we extend directional features from phase consistency to generate the directional gradient of omnidirectional phase consistency. We tested our method on 50 groups of images, comparing it with SIFT, SURF, ORB, HAPCG, and other techniques, with results demonstrating superior performance.

(1) The proposed algorithm effectively targets the weak texture characteristics of long-wave infrared images of the sea surface, improving matching accuracy. The mean NCM is 1147, the mean RMSE is 1.53, and the matching success rate is 100%, with minimal displacement differences.

(2) For mid-wave infrared images, the average NCM for two adjacent frames is 6727 with the proposed algorithm. In comparison, the average NCM for images with a 10-frame difference is 9756, the highest among all algorithms tested. The RMSE volatility remains stable, averaging 1.47. The algorithm demonstrates resilience against sea surface targets, facilitating better feature extraction from weak texture information.

(3) The proposed algorithm significantly improves rotation invariance in image matching, achieving effective results even with large rotation angles. The NCM value fluctuates around 1200, and the matching success rate remains high, with the RMSE staying below two pixels.

Previous research on matching algorithms for sea surface images has been relatively limited, primarily due to the highly dynamic nature of the sea surface and the complexity and variability of its environment. As such, matching sea surface images poses significant challenges. The method we propose offers both high accuracy and robustness. After performing the matching, the resulting fusion and stitching processes will significantly reduce artifacts and error rates, contributing greatly to improvements in the spatial and radiometric resolution of the stitched images. However, due to the rapid changes in the sea surface environment, even when using existing high-frame-rate infrared cameras for imaging, it is inevitable that matching errors will occur. Additionally, while the proposed method achieves good matching performance, it is undeniable that it takes more time compared to traditional algorithms. As a result, the method cannot process images in real time for practical applications and requires further improvements.

In conclusion, sea surface image-matching technology is rapidly advancing. Continuous innovation and research in various methods are significantly propelling this field forward. As data-acquisition technologies and algorithms evolve, sea surface image matching will increasingly play a critical role in diverse application scenarios.

## Figures and Tables

**Table 1:** Test results statistics of five algorithms.

Dataset	SIFT		SURF		ORB		HAPCG		Textual Algorithm	
	NCM	RMSE	NCM	RMSE	NCM	RMSE	NCM	RMSE	NCM	RMSE
A1	101	0.503	26	0.606	168	0.539	922	1.462	1309	1.473
A2	75	1.216	23	1.383	81	1.546	793	1.562	1056	1.632
A3	93	4.258	94	1.319	405	0.516	905	1.37	1224	1.442
A4	81	6.301	87	3.243	292	1.686	776	1.54	998	1.601
B1	4375	1.143	1470	24.347	12,291	1.872	7656	1.299	9794	1.295
B2	238	7.615	533	61.161	1557	7.893	4921	1.613	6831	1.651
B3	3939	1.126	1369	25.611	11,558	1.412	7362	1.319	9717	1.316
B4	222	7.532	481	60.313	1538	7.801	4662	1.632	6641	1.663

## Data Availability

The data that support the findings of this study are available from the corresponding author, [J.C.], upon reasonable request.
